# A Comprehensive Approach to Days’ Supply Estimation in a Real-World Prescription Database: Algorithm Development and Validation Study

**DOI:** 10.2196/83465

**Published:** 2026-02-11

**Authors:** Maria Malk, Kerli Mooses, Marek Oja, Johannes Holm, Hanna Keidong, Nikita Umov, Sirli Tamm, Sulev Reisberg, Jaak Vilo, Raivo Kolde

**Affiliations:** 1 Institute of Computer Science University of Tartu Tartu, Tartu Estonia; 2 Institute of Family Medicine and Public Health University of Tartu Tartu Estonia

**Keywords:** prescription records, days’ supply, medication adherence, CMA, continuous multiple interval measures of medication availability, daily dose, imputation

## Abstract

**Background:**

For accurate medication usage statistics and medication adherence calculations, we need to have an accurate days’ supply (DS) for each prescription. Unfortunately, often the DS or the information needed for calculating the DS is not provided. Therefore, other methods need to be applied to acquire missing values or substitute incorrect values.

**Objective:**

This study aims to apply a variety of methods for managing incomplete and missing data to enhance the accuracy of calculating DS for all medications and drug forms alike. Furthermore, to describe the effect of applied methods on the medication adherence calculated on real-world data.

**Methods:**

A dataset comprising prescription records from a 10% (150,824 patients) random sample of the Estonian population between 2012 and 2019 was used. The workflow consisted of 3 steps: data cleaning, imputation, and calculation of DS. For imputation, different methods were combined, such as calculating mode-based daily dose, or using usage guidelines from the Summary of Product Characteristics or legislation. DS was calculated based on the provided daily dose or imputed value. To evaluate the impact of data cleaning, medication adherence for the baseline dataset and corrected dataset for 2 time periods, 2012-2015 and 2017-2019, was calculated and compared.

**Results:**

The drug forms with the lowest proportion of correct DS provided were insulin injections (2601/82,867, 3.1%) and intravaginal contraceptives (1692/21,145, 8%) while the highest proportion of DS was provided for inhalation medication (78,541/126,588, 62%), oral drops (52,085/98,221, 53%) and tablets, capsules, suppositories (2,828,617/6,176,585, 45.8%). As a result of applying different imputation approaches, we successfully found the DS for 98.3% (7,415,347/7,544,892) of dispensed prescriptions. For the remaining 1.7% (129,545/7,544,892) of prescriptions, DS could not be imputed nor calculated with these methods. As for the medication adherence, the distinction between 2 observed time periods was more distinct in the baseline dataset compared with the corrected dataset for most of the drug groups, indicating that the applied correction methods had lessened the stark contrast.

**Conclusions:**

In summary, our study demonstrated that with a carefully designed imputation pipeline where data-driven imputation is combined with domain knowledge and literature information, it is possible to meaningfully improve the quality of prescription datasets and generate more accurate and consistent adherence metrics across various drug forms. Nonetheless, future efforts should continue to refine imputation techniques, incorporate machine learning approaches where appropriate, and expand validation efforts using external benchmarks or clinical outcomes.

## Introduction

Electronic health care databases provide a valuable data source for conducting various studies, as they are detailed, structured, and often cover a long time span. One important source of such data is pharmacy medication records, which provide the opportunity to research medication usage and adherence cost-effectively and at scale [1-3]. To calculate medication adherence, an accurate days’ supply (DS) for each prescription is needed [4]. DS describes how many days the dispensed medication is expected to last. In medical fields, DS is also referred to as treatment course length. While some prescription databases have DS recorded, in others, the DS value needs to be calculated using other available information, such as the number of dispensed medications and prescribed daily dose [4]. Unfortunately, there is an abundance of evidence suggesting inaccuracies and missing values in the prescription databases [4,5].

Several studies have specifically addressed the challenges related to missing daily doses and the DS issue [4,6,7]. For example, imputing 1 dose of medication per day for missing daily doses has been shown to work for patients with stroke [6], while imputing the mode daily dose per active substance and number of tablets per prescription has been shown to work for diabetic drugs [4]. Studies have also demonstrated that daily dose values can be imputed using machine learning (ML) algorithms that incorporate various patient characteristics [4]. Some studies [8,9] have applied the defined daily dose (DDD) toolkit developed by the World Health Organization [10]. However, it has been concluded that using DDD as a daily dose substitute may lead to misclassification of medication adherence [8,9]. The limitations of the existing studies tackling the missing daily dose and DS issue are that they often focus on 1 disease or medication group [4,6,7,9,11-13] and thus, it is unknown whether the same approach applies to other active substances or diseases. Moreover, studies have often been conducted using single-dose oral medications [4,6,7,9], and little is known about how to address the missing data in other drug forms such as eye drops, topical creams, and gels. Although some studies have researched medication adherence among diseases that often use other drug forms [11,13-18], only a few of these use pharmacy records [13,17]. However, these studies have not tackled the problem of missing data.

In addition to missing data, some studies have highlighted that some inaccuracies may be present in DS values [5,19]. More common inconsistencies are in reported DS values, dosage, fill intervals, administration times and quantity [5,19]. It has been stressed that further research is needed to evaluate DS reporting errors and to recommend strategies to address these errors [20].

To the best of our knowledge, no comprehensive approach exists that addresses both missing and inaccurate data in prescriptions database across all prescriptions, irrespective of the active substance or drug form. To address this shortcoming, this study aims to apply a variety of methods for managing incomplete and missing data to enhance the accuracy of calculating DS for all medications and drug forms alike. In addition, we describe the effect of applied methods on the medication adherence calculated on real-world data.

## Methods

### Data

The dataset used in this study consisted of prescription data for a 10% (150,824 patients) random sample of the Estonian population, covering the period from 2012 to 2019 [21]. The data originated from the national e-prescription database, where all prescriptions issued in primary and secondary care have been stored since 2010. The dataset includes all prescribed medications together with their dispensing information. Specific information about the prescriptions is shown in Table 1. Notably, the dataset does not contain information about over-the-counter medications or inpatient medications.

**Table 1:** Available prescription information.

Categories	Details
Patient information	Unique ID for each patient
General administrative information	Unique ID for each prescription Issue date Validity date Dispensed date Prescription type (initial, refill, narcotic, and medical device) Prescribing health care provider information Diagnosis code for which the medication was prescribed Rationale for brand-specific prescribing Dispensing pharmacy information Prescription cancellation reason
Active substance	Names of active substances Amount of maximum 3 components Unique ATC^a^ code for active substance
Drug form	Type of medication (eg, tablet, cream, and eye drops)
Daily dose information	Number of times the medication is taken or appliedb Amount of medication per doseb Optional free-text
Days’ supply	Optional treatment course length
Dispensed package information	Unique ID for each drug package Number of packages Number of units in each package Item size (for non-single-dose medications, eg, creams and eye drops)

^a^ATC: Anatomical Therapeutic Chemical.

These multiplied make up the daily dose.

In total, the dataset initially comprised 9,279,082 prescriptions, of which 7,544,892 (81.3%) were dispensed to patients. The rest of the prescriptions were prescribed but never dispensed. As it stands, only the prescriptions that were dispensed were included in this study and equipped with a DS value.

There were several data quality issues in the database that had to be addressed. Providing daily dose information became mandatory for doctors in mid-2016. As a result, a 67.4% (3,070,512/4,555,074) of prescriptions issued prior to this date lack daily dose information compared with 0.4% (12,468/2,989,805) of prescriptions issued in 2017-2019. Throughout this study period, it was optional for the doctors to specify the treatment course length for each prescription. This treatment course length is equivalent to the DS and could be used as a substitute or for comparison with the calculated DS. The number of prescriptions with provided DS increased over time–in 2012-2016, 15.2% (693,868/4,555,074) of prescriptions had this information compared with 36.1% (1,078,710/2,989,805) in 2017-2019, calculated in the respective timeframes.

Even though almost all prescriptions after 2016 were provided with a daily dose, the information was inconsistent in terms of the units used. The amount of medication could be given as a quantity (eg, 1 tablet, 2 pills, etc) or the amount of active substance (eg, 10 mg, 20 mg, etc). Neither of these writing methods was consistent among themselves either. For example, if a drug contained 2 active substances, like 5 mg + 10 mg, and the doctor decided to write the amount taken at once in active substance amount, they had 3 distinct ways to write that: 5 mg, 10 mg, and 15 mg.

For non–single-dose medications such as drops, creams, and gels, the daily dose was often noted only qualitatively (eg, “once per day”), without quantitative detail, making DS impossible to calculate.

### Workflow

**Figure 1:**
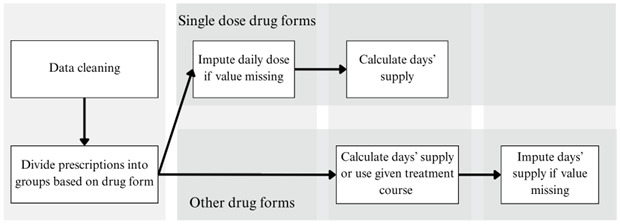
Workflow to acquire days’ supply.

The main workflow to acquire the DS value is presented in Figure 1.

### Data Cleaning and Drug Form Grouping

The prescriptions were first checked for correctness. Some of the prescriptions from 2012 had false active substance data. Since every dispensed medication has a package number, the correct active substance was taken from the package information.

Prescriptions were stratified by drug form into distinct groups, since each category follows specific conventions for stating the daily dose. For example, liquid formulations record daily doses in drops, creams in standardized dose units, and sprays in actuations. If needed, these groups were further divided by active substances because DS can be imputed more correctly for pharmacologically similar active substances and drug forms. For example, ophthalmic preparations were initially divided by drug form into 2 groups: single-dose containers and multiple-dose containers. The multiple-dose container group was further subdivided into 2 categories: one containing anti-infective, anti-inflammatory agents, and their combinations, and another containing all other substances. The last 2 subgroups required different imputation values.

All tablets, capsules, and suppositories share similar daily dose parameters, which allows for a consistent approach in calculating DS. These were collectively referred to as single-dose medications. Groups containing fewer than 1000 prescriptions were given a DS value based on the provided DS data and were excluded from further imputation processes.

### Single-Dose Drug Forms DS

For single-dose medications, if all the necessary daily dose information, consisting of the amount of medication in a dose and the number of doses in a time unit (ie, day or week), was available, we calculated the DS. Since there are many different ways to prescribe the medication amount in 1 dose, we used a method that took into consideration whether the amount of medication in a dose was written in units (1 tablet, 2 tablets, etc) or active substance amount (5 mg, 10 mg, etc). The number of doses per time unit, which is required for daily dose estimation, was generally accurate and therefore used without modification. Afterwards, we compared the calculated value with the provided DS, if it was available. Whichever of the 2—calculated or provided DS—had a smaller value, it was used. The rationale is that a pharmacist may dispense a larger package (ie, more tablets) if no package exactly matches the prescription. If the provided DS was not available, the calculated DS was used.

For prescriptions where DS could not be determined using the aforementioned logic, a mode-based imputation method was used. A reference dataset was created with the most common daily dose for each active substance and corresponding dose strength. When 2 or more daily dose values were equally frequent, those active substances were excluded from imputation, and their prescriptions remained unimputed. DS was then calculated for imputed prescriptions. This approach adds dose specificity to the active substance classification, making the DS value more reliable compared to direct DS imputation.

### Other Drug Forms DS

The general workflow for determining DS in the other dosage‐form groups was as follows. The target drug group was thoroughly analyzed to identify trends in how daily doses are recorded and the specific characteristics of its treatment regimens. Where possible, the DS was calculated, and the existence of the prescribed DS was checked. If prescribed DS information was available, it was either used directly or compared to the computed DS value, and the more appropriate of the 2 was adopted. This comparison was consistently applied and therefore not explicitly restated in the subsequent list.

In cases where neither of these approaches could be applied, alternative imputation strategies were used. These strategies were based on the Summary of Product Characteristics (SPCs) [22], where the recommended DS is written and on relevant Estonian regulations [23], where, for example, the recommended prescription usage length is recorded. These outcomes were then reviewed by a domain expert and, if necessary, adjusted to reflect current practices in the field. Based on this evaluation, specific imputation rules were defined for each medication group and subsequently applied in the processing workflow.

More specifically, the DS was calculated or imputed for different drug forms as follows:

1. For semisolid medications consisting of creams, gels, shampoos, and ointments for topical usage, daily dosing instructions are often provided without quantitative details. Consequently, implementing a DS calculation method would give limited value and was therefore not applied. If the provided DS was available, that value was used. Otherwise, it was imputed as 30 days per package.
2. For medicinal nail polish, daily dosing instructions are often provided without quantitative details. Therefore, DS calculations were not done. When the provided DS was available, it was used. Otherwise, DS was imputed based on the package type. As only 2 package types were present in the dataset, the DS was either 210 days or 180 days.
3. For eye drops, daily dosing instructions are often provided without quantitative details. Therefore, the DS calculation was not done. If the provided DS was available, it was used. Otherwise, due to shelf-life limits, DS was imputed as 30 days per package, except for anti-infectious, anti-inflammatory, and corticosteroid drops, which should not exceed a 2-week treatment course. Therefore, DS were imputed with either 30 days or 14 days, depending on the active substance. When the eye drops were packaged in a single-dose container, and a daily dose was provided, the daily dose was used to calculate DS. If daily data were not available, the provided DS was used, and if neither was available, it was imputed as 30 days.
4. For ear drops, all medications present in the dataset were for short-term use only. Namely, anti-infective and analgesic medications, which are usually not used for more than 7 days without a doctor’s supervision. Since the daily dosing instructions are often provided without quantitative details, the DS calculations were not done. When the provided DS was available, it was used. Otherwise, a 7-day value was used for imputation.
5. For oral drops, if the daily dose was specified quantitatively (eg, in number of drops or amount of active substance), this information was used to calculate the DS. The calculations varied depending on the active substance, as the drop sizes differed between substances. When the DS could not be calculated, and provided DS was available, it was used. Otherwise, 30 days per package was imputed due to shelf-life limits.
6. For nasal sprays, DS was calculated based on the daily dose if it was provided. If the DS could not be calculated, we used the provided DS if it was available. Otherwise, a default imputed value of 30 days per package was applied except for the antifungal nasal spray, where the treatment course should not last more than 7 days. Therefore, for imputation, the 7-day value was used.
7. For inhalation powders, if the medication was divided into blisters and a daily dose was provided, then it was used to calculate DS. When the DS could not be calculated, and provided DS was available, it was used. Otherwise, a value of 60 days was imputed for all prescriptions, considering the prescription guidelines and prevailing trends. According to Estonian legislation and prescribing practices, chronic medications are issued on a single prescription for a 2-month period.
8. For syrups, if the daily dose was specified quantitatively, it was used to calculate the DS. When the DS could not be calculated, and provided DS was available, it was used. Otherwise, 30 days per package was imputed due to shelf-life limits.
9. For antibiotic solutions, daily dosing instructions are often provided without quantitative details, therefore DS calculation was not done. When the provided DS was available, it was used. For these medications, the treatment course should not be longer than 14 days; therefore, for imputation 14-day value was used.
10. For transdermal patches, daily dosing instructions are often provided without quantitative details, therefore DS calculation was not done. When the provided DS was available, it was used. Otherwise, the imputable DS was calculated based on the length of the effect of the patches based on information from SPCs.
11. For vaccines and implants, the treatment course was imputed as 1 day. Since there is no standard way for doctors to write a DS value, it was not used.
12. For insulin, a value of 60 days was imputed for all prescriptions, considering the prescription guidelines and prevailing trends. According to Estonian legislation and prescribing practices, chronic medications are issued on a single prescription for a 2-month period.

### Quality Control Using Medication Adherence

To assess the impact of data cleaning and imputation, we calculated the mean value of DS together with 95% CI values for 147 active substances in the corrected datasets used in chronic conditions for 2 time periods: 2012-2015, when providing daily dose information was voluntary and 2017-2019, when this information was mandatory for the doctors. Since 2016 was a transition year, it was excluded from the time-period comparison. This approach shows us whether imputation introduced systematic bias in prescription duration. Moreover, we calculated medication adherence for these active substances in both the baseline and corrected datasets. This approach provides a broader statistical perspective across all medications, allowing us to evaluate the validity of the imputations based on all the prescriptions available.

The active substances used in the analysis were selected as follows: 300 of the most frequently prescribed active substances were extracted from the database, and 2 pharmacists independently filtered out those that were meant for chronic conditions. The active substances used in calculations were divided into 27 groups based on the Anatomical Therapeutic Chemical (ATC) therapeutic subgroup (Multimedia Appendix 1).

In the baseline dataset, the DS was calculated based on the provided daily dose for single-dose medications. When the DS was not provided, a value of 30 days was imputed [4]. Baseline dataset analyses were performed using a simplified approach, which is fast, inexpensive, and requires minimal data processing. In the corrected dataset, we applied more resource-intensive methods to evaluate whether such refinement provides a meaningful advantage in adherence estimation.

For medication adherence calculations, the continuous multiple interval measures of medication availability (CMA) [2] were used. Out of 8 CMAs, CMA5 was selected as it accounts for gaps in medication availability and assumes that the new refill is stored until the previous prescription is fully used. The adherence was calculated on a yearly basis. The calculation window began with the first medication dispensing and ended with the last, requiring each patient to have at least 2 prescriptions dispensed. Any unused medication remaining at the end of the window was excluded from the calculations [2]. The CMA implementation in AdhereR [24] was used through AdherenceFromOMOP [25].

To describe the change in medication adherence between 2 periods (2012-2015 vs 2016-2019), the change in period means was calculated. Although the year 2016 was a transition year and excluded from the time-period comparison, the data for 2016 are presented in CMA figures in Multimedia Appendix 1.

### Ethical Considerations

The study was approved by the Research Ethics Committee of the University of Tartu (300/T-23), the Estonian Committee on Bioethics and Human Research (1.1-12/653), and the requirement for informed consent was waived.

## Results

A total of 7,544,892 dispensed prescriptions were included in the process of establishing the DS value. The largest drug form group was single-dose medications, including tablets, capsules, and suppositories, which accounted for 81.9% (n=6,176,585/7,544,892) of all dispensed prescriptions. The remaining drug form groups are listed in Table 2. In total, 13 major drug form categories were identified.

**Table 2:** Prescription distribution by drug groups and days’ supply establishing methods, for the full dataset (2012-2019).

Drug group and dataset information			Method for finding days, supply		Prescriptions in main group, n (%)
**Tablets, capsules, and suppositories**					
	Daily dose provided	Calculated using the given daily dose		2,828,617 (45.80)	
	Days’ supply provided	Days’ supply was used		933,710 (15.12)	
	Neither daily dose nor days’ supply provided	Calculated using the imputed daily dose value		2,402,392 (38.89)	
	Days’ supply could neither be calculated nor imputed	—^a^		11,866 (0.19)	
**Semisolid dosage forms**					
	Days’ supply provided	Days’ supply was used		119,748 (37.55)	
	Days’ supply not provided	Imputed as 30 days per package^b^		199,154 (62.45)	
**Medicinal nail polish**					
	Days’ supply provided	Days’ supply was used		3008 (41.55)	
	Days’ supply not provided	Imputed as 210 days or 180 days^b^		4231 (58.45)	
**Ear drops**					
	Days’ supply provided	Days’ supply was used		5661 (57.11)	
	Days’ supply not provided	Imputed as 7 days per prescription^b^		4252 (42.89)	
**Eye drops**					
	Eye drops in single-dose containers - daily dose provided	Calculated using the given daily dose		6724 (2.35)	
	Other eye drops - days’ supply provided	Days’ supply was used		62,332 (21.81)	
	Other eye drops - days’ supply not provided	Imputed as 14^b^ or 30^c^ days per package		216,740 (75.82)	
	Days’ supply not calculated nor imputed	—		43 (0.02)	
**Oral drops**					
	Daily dose provided	Calculated using the given daily dose		52,085 (53.03)	
	Days’ supply provided	Days’ supply was used		33,055 (33.65)	
	Neither daily dose nor days’ supply provided	Imputed as 30 days per prescription^b^		12,961 (13.20)	
	Days’ supply neither calculated nor imputed	—		120 (0.12)	
**Inhalation medication**					
	Daily dose provided	Calculated using the given daily dose		78,541 (62.04)	
	Days’ supply provided	Days’ supply was used		19,885 (15.71)	
	Days’ supply not provided	Imputed as 60 days per prescription^b^		27,137 (21.44)	
	Days’ supply not calculated nor imputed	—		1025 (0.81)	
**Nasal sprays**					
	Antifungal nasal spray - days’ supply not provided	Imputed as 7 days per prescription^b^		7738 (8.72)	
	Daily dose provided	Calculated using the given daily dose		26,361 (29.70)	
	Days’ supply provided	Days’ supply was used		40,551 (45.69)	
	Neither daily dose nor days’ supply provided	Imputed as 30 days per prescription^b^		14,094 (15.88)	
**Syrups**					
	Antibiotics - days’ supply not provided	Imputed as 14 days per prescription^b^		29,130 (34.31)	
	Daily dose provided	Calculated using the given daily dose		37,251 (43.87)	
	Days’ supply provided	Days’ supply was used		3094 (3.64)	
	Days’ supply not provided	Imputed as 30 days per package^c^		15,436 (18.18)	
**Transdermal patch**					
	Hormonal patch - days’ supply not provided	Impute as 7 days per patch plus 7 days^b^		13,131 (81.18)	
	Analgesic patch - days’ supply not provided	Imputed as 3 days per patch^b^		1127 (7.01)	
	Days’ supply provided	Days’ supply was used		599 (5.94)	
	Days’ supply not provided	Impute as 7 days per patch^b^		865 (5.38)	
**Intravaginal contraceptives**					
	Days’ supply provided	Days’ supply was used		1692 (8)	
	Days’ supply not provided	Imputed as 30 days per item in package^b^		19,453 (92)	
**Implants and vaccines**					
	Days’ supply provided	Days’ supply was used		18,940 (45.2)	
	Days’ supply not provided	Imputed as 1 day		22,979 (54.8)	
**Insulin injections**					
	Days’ supply provided	Days’ supply was used		2601 (3.14)	
	Days’ supply not provided	Imputed as 60 days per prescription^b^		80,266 (96.86)	
**Others**					
	Days’ supply provided	Days’ supply was used		69,806 (37.47)	
	Days’ supply not calculated nor imputed	—		116,491 (62.52)	

^a^Not available.

^b^Based on the Summary of Product Characteristics issued by the Estonian Agency of Medicines.

^c^Medication not shelf-stable for more than 30 days after opening.

The drug forms with the lowest proportion of correct DS provided were insulin injections (2601/82,867, 3.1%) and intravaginal contraceptives (1692/21,145, 8%) while the highest proportion of DS was provided for inhalation medication (n=78,541/126,588, 62%), oral drops (52,085/98,221, 53%) and tablets, capsules, suppositories (2,828,617/6,176,585, 45.8%), in their respective drug form group (Table 2).

For tablets, capsules, and suppositories, 38.9% (2,402,392/6,176,585) of prescriptions lacked daily dose information and were therefore imputed using the mode-based imputation described earlier. The mode table consisted of 1002 active substances and dosage amount combinations, out of which 60.1% (602/1002) of combinations had the value of once per day, and 24.7% (245/1002) of these combinations had the value of twice per day. In cases where both a calculated DS and provided DS information were available, the smaller value was used out of the 2. As a result, 15.1% (933,710/6,176,585) of prescriptions in single-dose medications were assigned DS provided by the doctor. For other drug forms, when the DS could not be calculated, instead of using the daily dose, we imputed the DS based on the specific drug form. The proportion of imputed prescriptions ranged from 13.2% (12,961/98,221) for oral drops to 92% (19,453/21,145) for intravaginal contraceptives and 96.9% (80,266/82,867) for insulin injections (Table 2; Multimedia Appendix 2). As a result of applying different imputation approaches, we successfully found the DS for 98.3% (7,415,347/7,544,892) of dispensed prescriptions. For 1.7% (129,545/7,544,892) of prescriptions, DS could neither be imputed nor calculated.

To evaluate the impact of data cleaning and imputation, the mean DS values and medication adherence of 147 active substances belonging to 27 ATC therapeutic subgroups were calculated.

**Figure 2:**
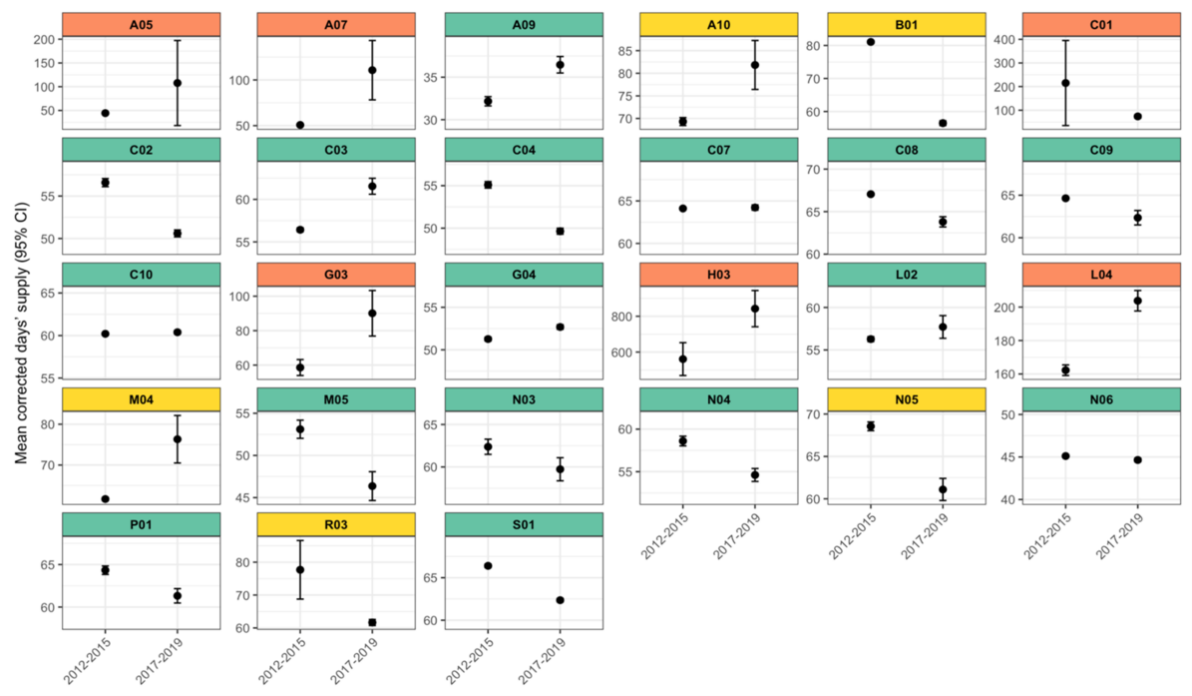
The mean value of DS and 95% CIs for a period where DS was mostly imputed (years 2012-2015) and mostly calculated based on data provided by doctors (years 2017-2019) by Anatomical Therapeutic Chemical subgroups. Green: ≤7 mean difference; yellow: 7-30 mean; red: > 30 mean difference.A05: bile and liver therapy; A07: antidiarrheals, intestinal anti-inflammatory and anti-infective agents; A09: digestives, including enzymes; A10: drugs used in diabetes; B01: antithrombotic agents; C01: cardiac therapy; C02: antihypertensives; C03: diuretics; C04: peripheral vasodilators; C07: beta blocking agents; C08: calcium channel blockers; C09: agents acting on the renin-angiotensin system; C10: lipid modifying agents; G03: sex hormones and modulators of the genital system; G04: urologicals; H03: thyroid therapy; L02: endocrine therapy; L04: immunosuppressants; M04: antigout preparations; M05: drugs for treatment of bone diseases; N03: antiepileptics; N04: anti-Parkinson drugs; N05: psycholeptics; N06: psychoanaleptics; P0: antiprotozoals, R03: drugs for obstructive airway diseases; S01: ophthalmologicals.

Comparison of the mean DS values between the period where most DS were imputed (2012-2015) and the period with more complete daily dose and DS data (2017-2019) revealed that the observed differences in mean DS were generally small and, in most cases, did not exceed the difference of 7 days (Figure 2). This indicates a high level of consistency between imputed and calculated values. Nonetheless, some ATC groups with larger deviations were present, for example, thyroid therapy (H03) and cardiac therapy (C01).

**Figure 3:**
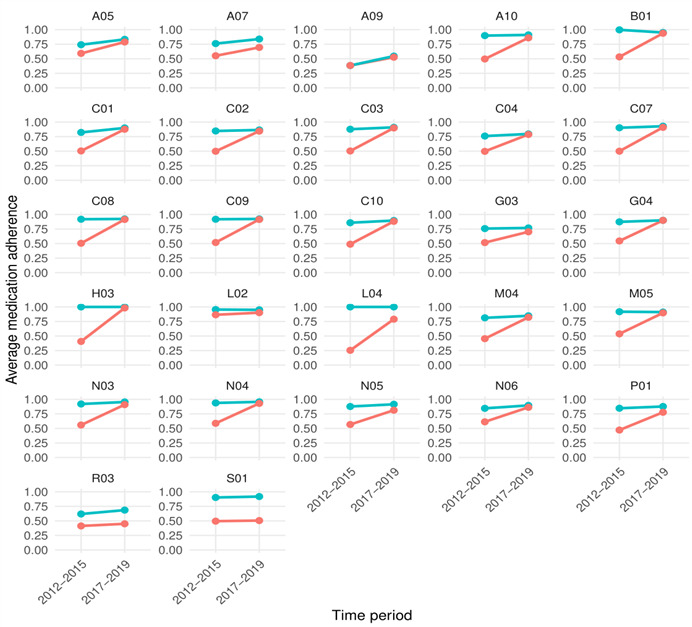
The average medication adherence by time period and Anatomical Therapeutic Chemical subgroups. Blue: corrected dataset; red: baseline dataset. A05: bile and liver therapy; A07: antidiarrheals, intestinal anti-inflammatory and anti-infective agents; A09: digestives, including enzymes; A10: drugs used in diabetes; B01: antithrombotic agents; C01: cardiac therapy; C02: antihypertensives; C03: diuretics; C04: peripheral vasodilators; C07: beta blocking agents; C08: calcium channel blockers; C09: agents acting on the renin-angiotensin system; C10: lipid modifying agents; G03: sex hormones and modulators of the genital system; G04: urologicals; H03: thyroid therapy; L02: endocrine therapy; L04: immunosuppressants, M04: antigout preparations; M05: drugs for treatment of bone diseases; N03: antiepileptics; N04: anti-Parkinson drugs; N05: psycholeptics; N06: psychoanaleptics; P01: antiprotozoals; R03: drugs for obstructive airway diseases; S01: ophthalmologicals.

The medication adherence comparison shows that for most of the drug groups, the difference between 2 observed time periods was more distinct in the baseline dataset compared with the corrected dataset, indicating that the applied correction methods had lessened the stark contrast (Figure 3; Multimedia Appendix 1). For the multiple-dose medications, such as drugs for obstructive airway diseases (R03) and ophthalmologicals (S01), there was no distinction between the observed time periods. However, the medication adherence improved similarly for both time periods. At the same time, some adherence measures, such as the thyroid therapy (H03), showed 100% of medication adherence.

## Discussion

### Principal Results

Accurate estimation of DS is essential for assessing medication adherence and conducting pharmacoepidemiological research. This paper set out to improve the completeness and precision of prescription data. We developed and implemented a multistep data cleaning and imputation approach to address missing or incomplete information in prescription records, specifically targeting the derivation of DS. Unlike previous studies, which typically focused on a single drug class or drug form [4,6-9,11,13-18,20,26,27], our work aimed to determine DS values for all prescriptions. By applying a combination of rule-based calculations, statistical imputation, and domain knowledge, we were able to assign DS values to almost all of the 7.5 million dispensed prescriptions included in this study dataset. This demonstrates the feasibility of using hybrid methods in large-scale, real-world prescription datasets, particularly when working with data mapped to standardized models.

The mean value comparison indicates a high level of consistency between imputed and observed values in most medication groups. These findings suggest that the imputation strategy provides a reasonable approximation of real-world prescription lengths and preserves the overall patterns in medication use. Nonetheless, isolated ATC groups with larger deviations show that imputed values may still under- or overestimate treatment duration in specific cases. Furthermore, it is evident that the imputation process did not systematically shift the DS estimates upward or downward. This suggests that the observed differences in DS between time periods might also be driven by external factors, such as changes in available package sizes or evolving prescribing practices.

The medication adherence calculated on the baseline database and corrected database suggests that our approach significantly improved the adherence estimates. For most drug groups, it became evident that once imputation methods were applied, the adherence estimates for the 2012-2015 period aligned more closely with those from 2017-2019, when daily dose reporting became mandatory for physicians and the overall database quality improved. At the same time, the adherence values for the 2017-2019 period for both datasets remained similar for most drug classes. This supports the hypothesis that earlier data underrepresented medication availability due to incomplete documentation, and that our imputation procedures improved temporal consistency and data reliability.

Some kind of imputation was needed in each observed drug class. The predominance of tablets, capsules and suppositories in the dataset enabled the use of daily dose–based imputations for a substantial portion of the records. Imputing 1 dose per day has proven to be an effective way to address the missing data for some oral drugs in a previous study with heart medications [6]. Although this approach was also applicable in our dataset, the mode DS values revealed that such a uniform approach was unsuitable for 39.9% (400/1002) of single dose medications imputation values. Therefore, using the mode-table imputation method helped to identify the most common daily doses per active substance and use this information in DS calculations. Although our approach significantly improves the data quality, it is not flawless. For example, the medication adherence calculated for thyroid therapy (H03) on the corrected database was 100%. Thus, raising the suspicion that it might be overestimated and the imputed daily doses were too small. It could be hypothesized that for some medications more than others, the individual treatment regimens may differ, and therefore, it is difficult to identify the most common dose to impute. When the mode value used in imputation is lower than the next most popular value, then it can result in a higher DS value and, therefore, better medication adherence values.

For non–single-dose drug forms, such as creams, drops, and gels, imputation based on SPCs and provided DS proved useful, though these methods are inherently less precise due to variability in usage patterns and dosing recommendations. In 2 drug classes, obstructive airway diseases (R03) and ophthalmologicals (S01), the medication improved for both time periods. One reason for this could be that in the baseline dataset, a rough imputation of 30 days was applied to all missing daily doses, while in this study, the imputed DS was more active substance specific and based on national SPCs.

In addition to imputing missing data, there is sometimes a question of the plausibility of provided DS that are given by a doctor with the prescription [5,19]. The question arises whether and how we assess the DS prescribed by physicians, and whether this should be compared with the calculated duration. Until 2016, our prescription system allowed manual entry of the duration, and errors can often occur. The most common and noticeable errors occurred when repeat prescriptions were issued, and each was assigned a DS of 180 days, which in fact represents the combined length of 3 prescriptions. This stems from the common practice of doctors providing 3 refill prescriptions per medication. These kinds of problems were easy to notice and fix, but more complex errors were harder to detect. Another example concerns vaccines and implants, for which no standard exists for indicating the DS on the prescription, and each doctor records it according to their own discretion. As a result of incorrect entries, some prescriptions may end up with an inaccurate DS value. Despite these challenges, our imputation methods combined with domain knowledge ensured reasonable estimates of DS across diverse drug forms.

Our study also underscores the importance of a user design and information architecture of the prescription database. It clearly emerged from this study that before the summer of 2016, when there was no requirement to record dosing instructions for medications, the data quality was lower and different methods were needed to backfill this information retrospectively. Therefore, to collect accurate data, more effort should be paid to the architecture of the system to ensure that all necessary data will be inserted and stored as correctly as possible. Moreover, there is a need to raise awareness among doctors on the importance of data quality and its effect on evaluating health care services and medication adherence. For example, we identified in the dataset that sometimes there is an inconsistency in the units of prescribed medications within prescriptions issued by doctors—one might use units (eg, 1 tablet) on one prescription and the amount of active substance (eg, 10 mg) on another. Moreover, some doctors have a practice to renew the old prescription without changing the dosing information, even when the dosing regimen changes. This all impacts the data quality, as detecting such cases from the data is very difficult, if not impossible. Therefore, beyond seeking sophisticated imputation methods to address the missing data, we should also consider improving the prescription systems and informing and educating the doctors who enter this data. More complete and accurate records would provide a better foundation for secondary use of prescription data in the future.

### Limitations

One limitation of our study is that there was no golden standard or reference database to compare the results with. Although the amount of missing data substantially reduced in the period 2017-2019 due to the changes in the prescription system, some inaccuracies due to the human component remained. However, it could be argued that the baseline data from 2017-2019 gives a considerably good indication of actual prescription patterns.

It is also important to acknowledge that imputation techniques possess inherent limitations and may not invariably produce fully accurate estimates. To construct a mode table of daily doses, a certain proportion of presumably correct prescription data is required; otherwise, it cannot be compiled. Furthermore, in some drug classes where the dosing recommendations vary based on the severity of disease, the mode table did not seem to be the best approach, as the medication adherence calculated on the corrected database was unrealistically high. Potentially, some ML methods could be more effective in such cases where mode tables fail, but this warrants further investigation. Moreover, ML methods should also be applied to other injections and less prevalent drug forms, which in this study were excluded.

### Conclusions

In summary, our study demonstrated that with a carefully designed imputation pipeline where data-driven imputation is combined with domain knowledge and literature information, it is possible to meaningfully improve the quality of prescription datasets and generate more accurate and consistent adherence metrics across various drug forms. Nonetheless, future efforts should continue to refine imputation techniques, incorporate ML approaches where appropriate, and expand validation efforts using external benchmarks or clinical outcomes.

## Data Availability

The datasets generated or analyzed during this study are not publicly available due to legal restrictions on sharing deidentified data. According to legislative regulation and data protection law in Estonia, the authors cannot publicly release the data received from the health data registries in Estonia. However, the data can be requested by completing necessary applications in order to carry out research or an evaluation of public interest and acquiring the permission of the controller of the databases.

## Appendix

Multimedia Appendix 1 Graphs of yearly medication adherence (2012-2019) showing baseline dataset and corrected dataset. Stratified by ATC code.

Multimedia Appendix 2 Prescription distribution by drug groups and days’ supply establishing methods, for the full dataset (2012-2019).

## Abbreviations

ATC: Anatomical Therapeutic Chemical
DDD: defined daily dose
DS: days’ supply
CMA: continuous multiple interval measures of medication availability
ML: machine learning
SPC: Summary of Product Characteristics
