# Supplementary material for: A Comprehensive Approach to Days’ Supply Estimation in a Real-World Prescription Database: Algorithm Development and Validation Study
Source: Online J Public Health Inform. 2026 Feb 11;18:e83465. doi: 10.2196/83465 (PMC12936656; doi:10.2196/83465)
Supplement: Multimedia Appendix 1 [file ojphi_v18i1e83465_app1.pdf]

Figure S1: Graphs of yearly medication adherence (2012–2019) showing baseline dataset and corrected dataset. Stratified by ATC code.

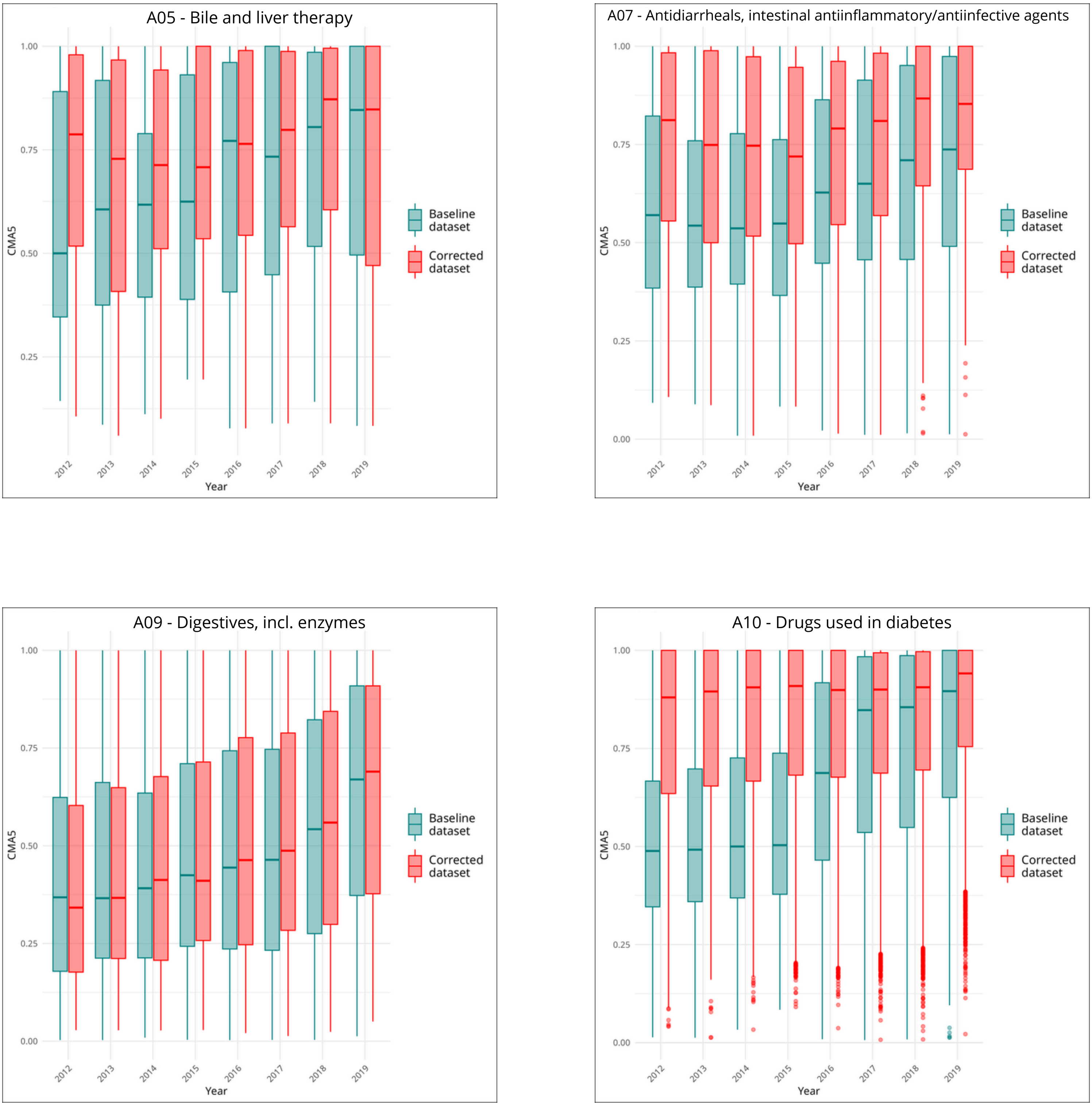

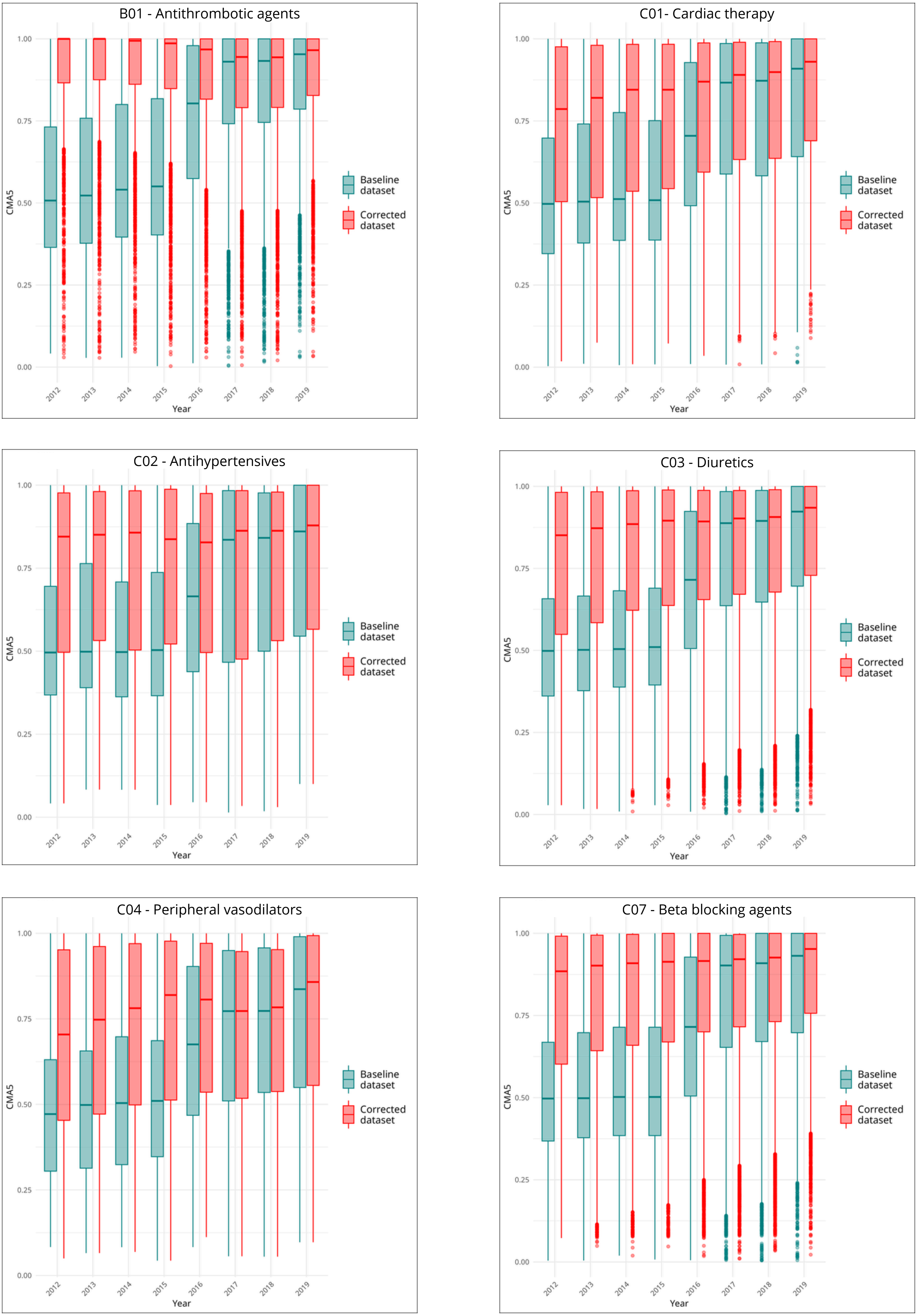
B01 - Antithrombotic agents
C01- Cardiac therapy
C02 - Antihypertensives
C03 - Diuretics
C04 - Peripheral vasodilators
C07 - Beta blocking agents
CMA5
0.00
0.25
0.50
0.75
1.00
Year
2012
2013
2014
2015
2016
2017
2018
2019
Baseline dataset
Corrected dataset

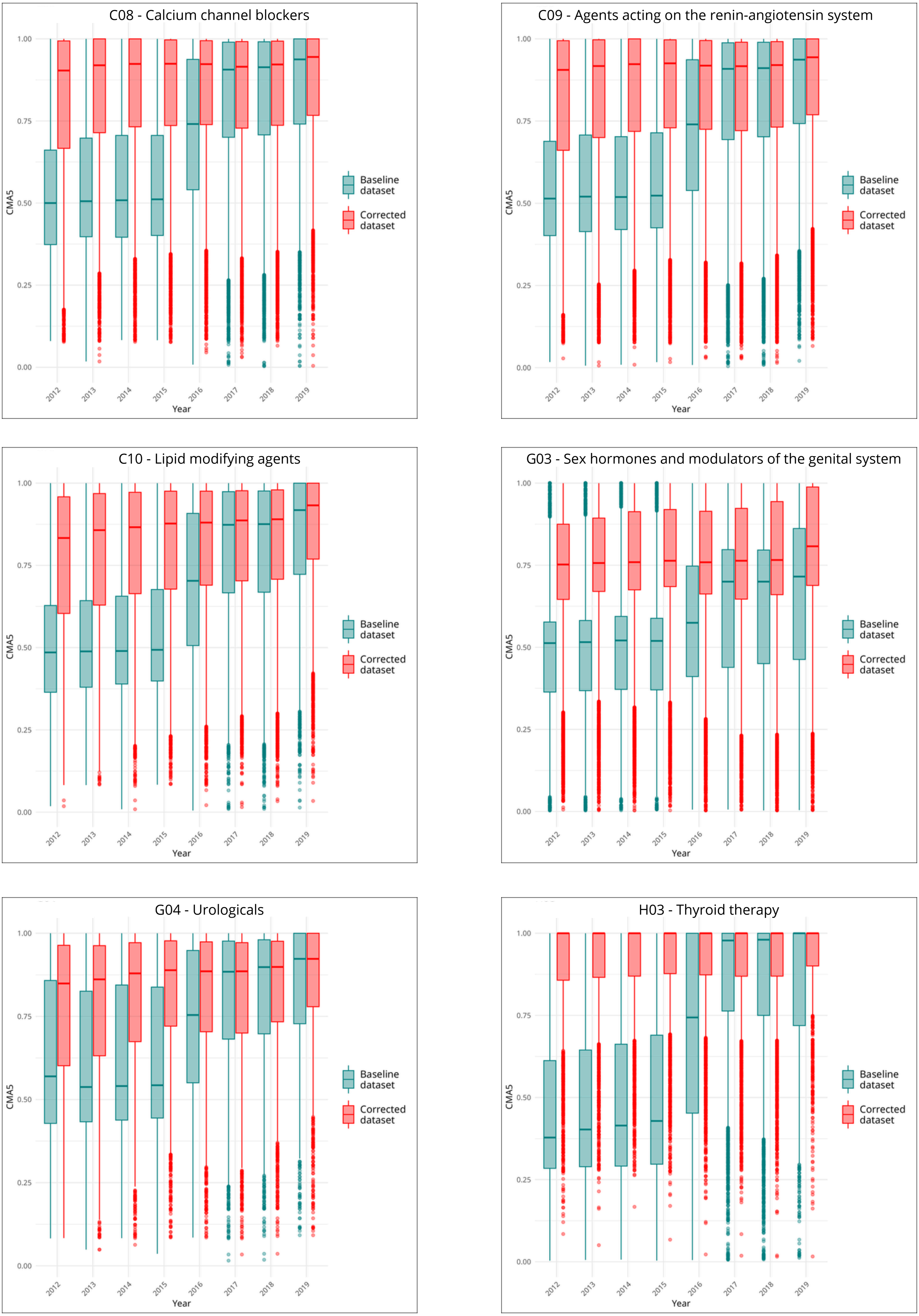
C08 - Calcium channel blockers
C09 - Agents acting on the renin-angiotensin system
C10 - Lipid modifying agents
G03 - Sex hormones and modulators of the genital system
G04 - Urologicals
H03 - Thyroid therapy
CMA5
Year
0.00
0.25
0.50
0.75
1.00
2012
2013
2014
2015
2016
2017
2018
2019
Baseline dataset
Corrected dataset

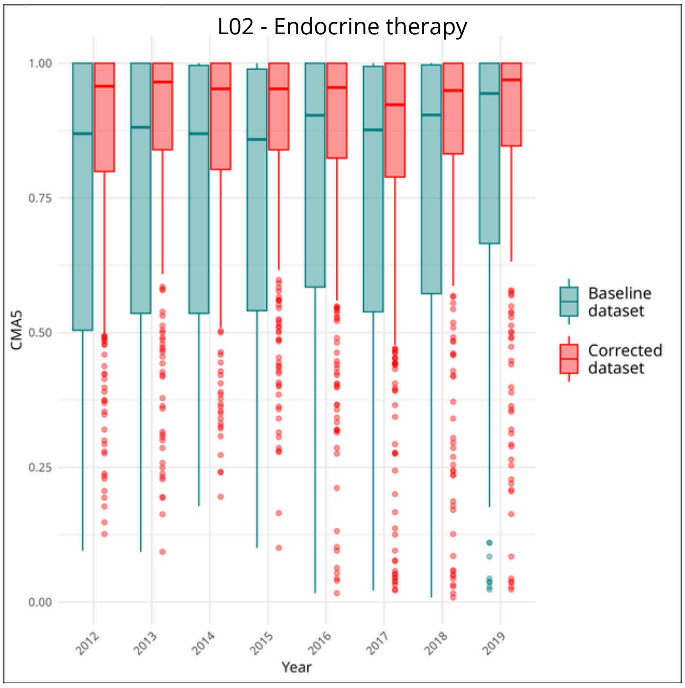
L02 - Endocrine therapy
CMA5
1.00
0.75
0.50
0.25
0.00
2012
2013
2014
2015
2016
2017
2018
2019
Year
Baseline dataset
Corrected dataset

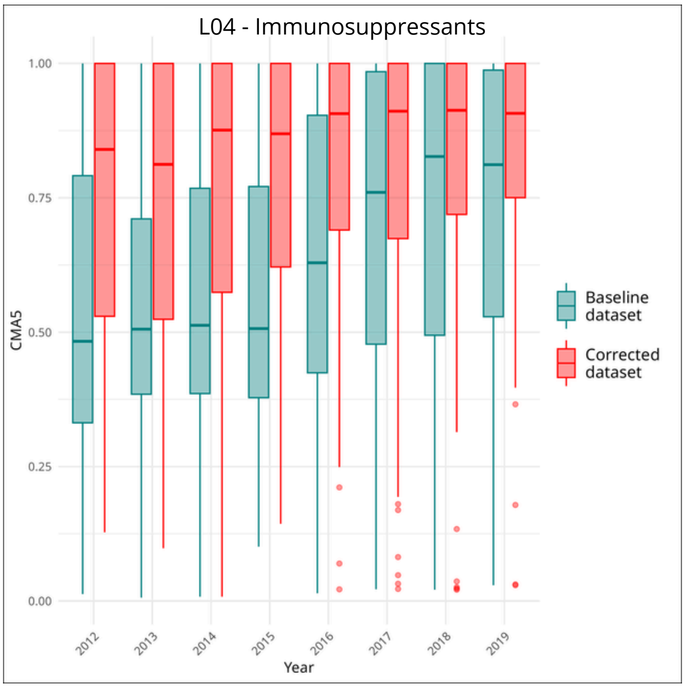
L04 - Immunosuppressants
CMA5
1.00
0.75
0.50
0.25
0.00
2012
2013
2014
2015
2016
2017
2018
2019
Year
Baseline dataset
Corrected dataset

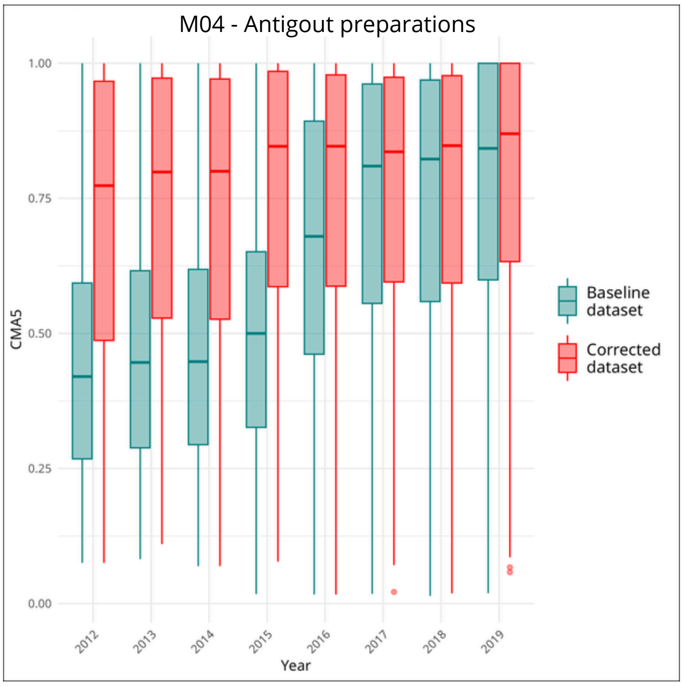
M04 - Antigout preparations
CMA5
1.00
0.75
0.50
0.25
0.00
2012
2013
2014
2015
2016
2017
2018
2019
Year
Baseline dataset
Corrected dataset

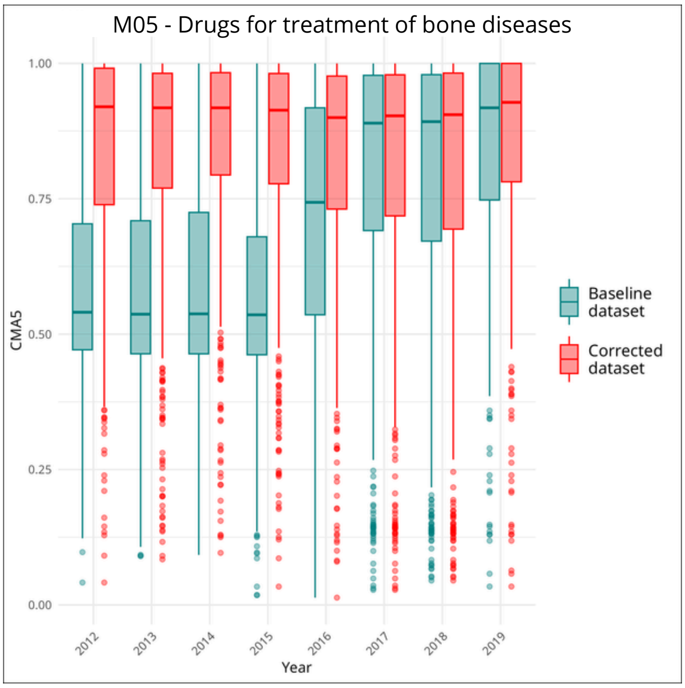
M05 - Drugs for treatment of bone diseases
CMA5
1.00
0.75
0.50
0.25
0.00
2012
2013
2014
2015
2016
2017
2018
2019
Year
Baseline dataset
Corrected dataset

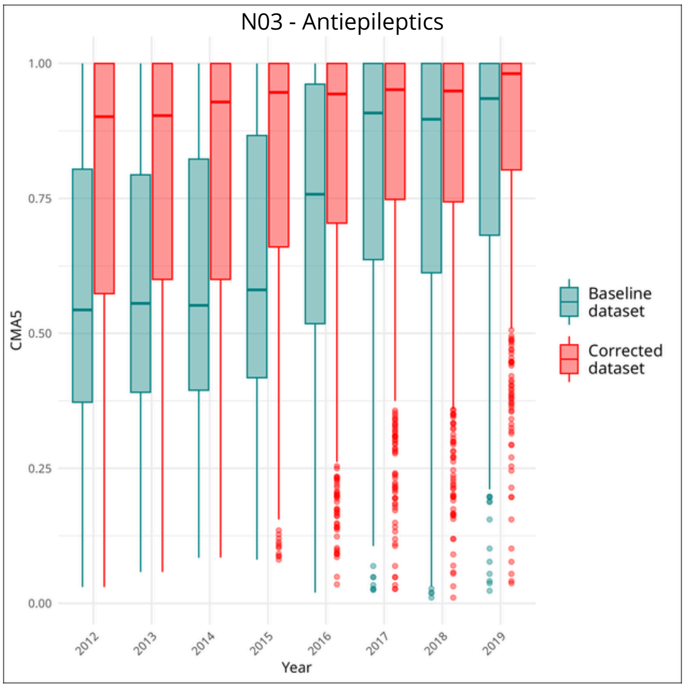
N03 - Antiepileptics
CMA5
1.00
0.75
0.50
0.25
0.00
2012
2013
2014
2015
2016
2017
2018
2019
Year
Baseline dataset
Corrected dataset

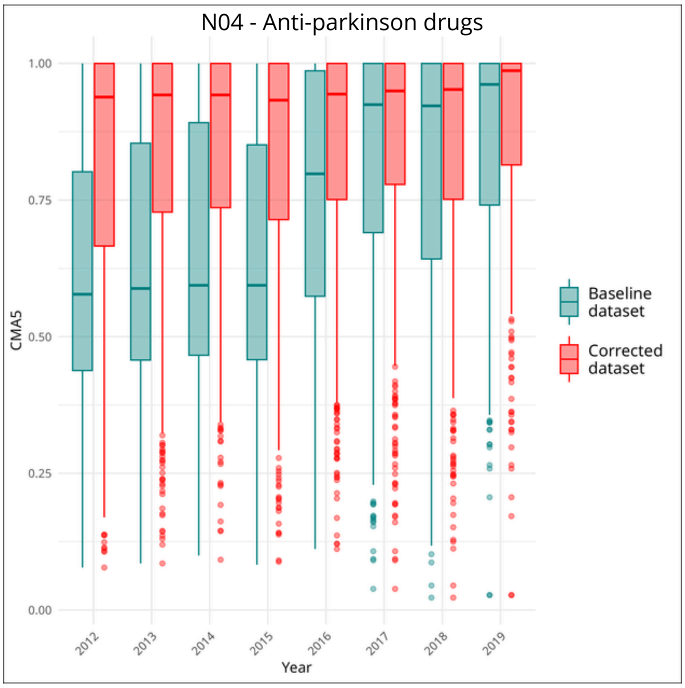
N04 - Anti-parkinson drugs
CMA5
1.00
0.75
0.50
0.25
0.00
2012
2013
2014
2015
2016
2017
2018
2019
Year
Baseline dataset
Corrected dataset

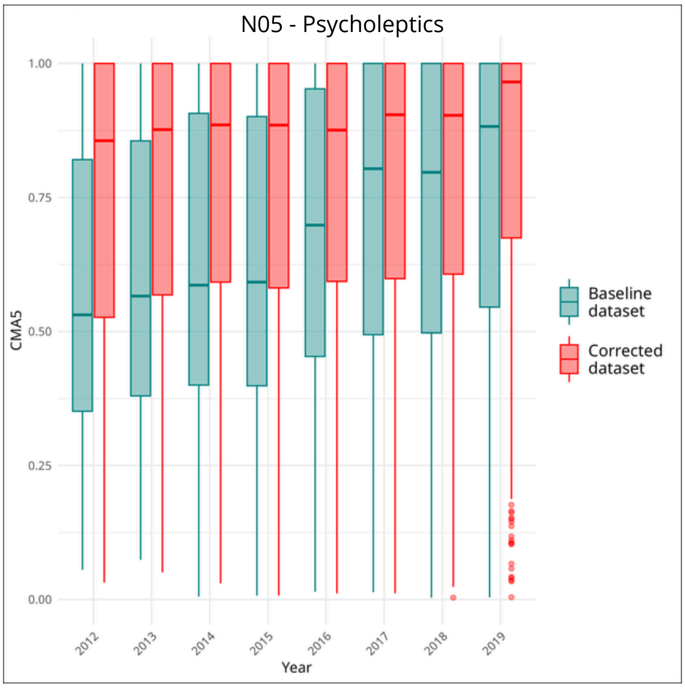
N05 - Psycholeptics
1.00
0.75
0.50
0.25
0.00
CMA5
2012
2013
2014
2015
2016
2017
2018
2019
Year
Baseline dataset
Corrected dataset

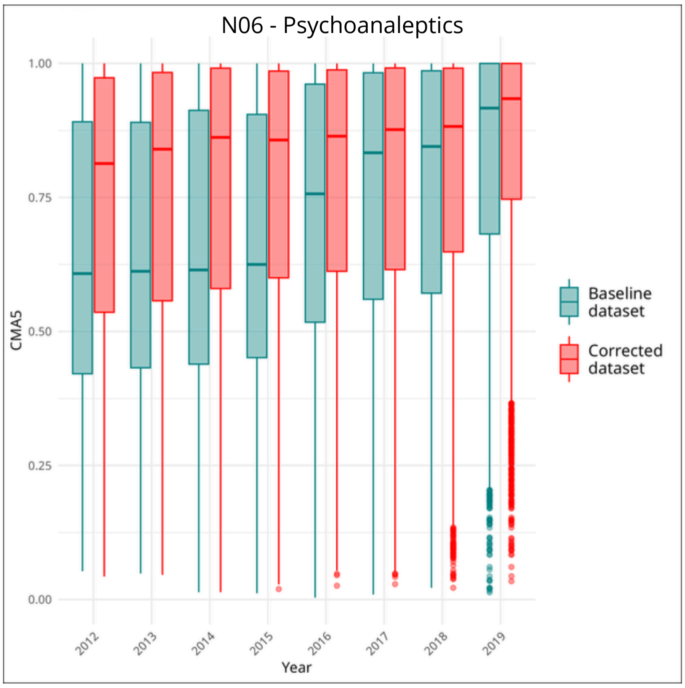
N06 - Psychoanaleptics
1.00
0.75
0.50
0.25
0.00
CMA5
2012
2013
2014
2015
2016
2017
2018
2019
Year
Baseline dataset
Corrected dataset

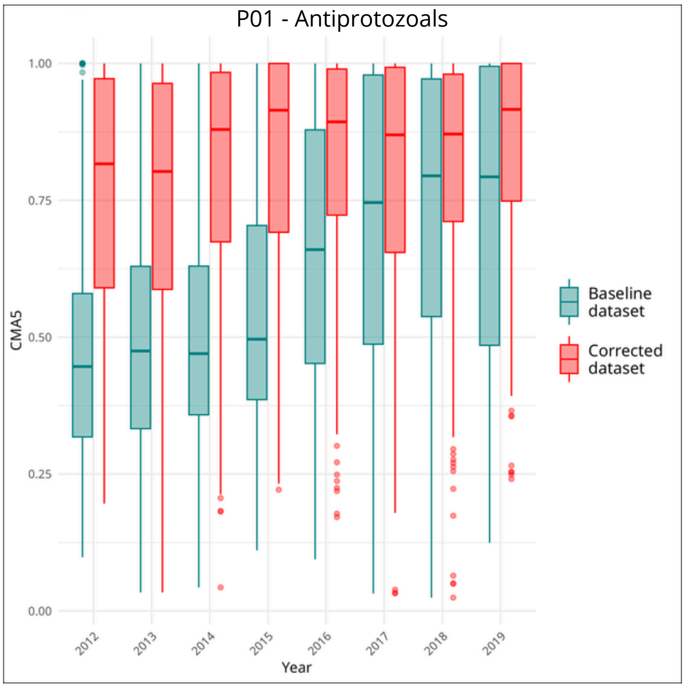
P01 - Antiprotozoals
1.00
0.75
0.50
0.25
0.00
CMA5
2012
2013
2014
2015
2016
2017
2018
2019
Year
Baseline dataset
Corrected dataset

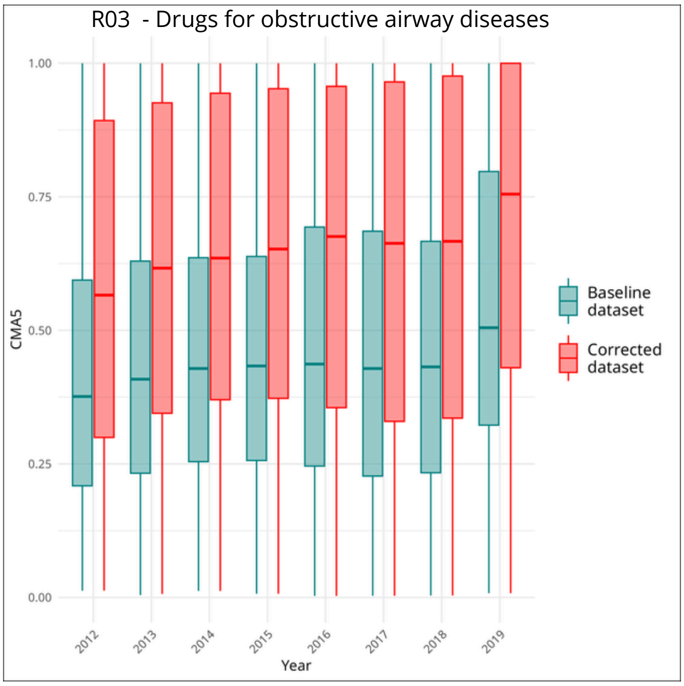
R03 - Drugs for obstructive airway diseases
1.00
0.75
0.50
0.25
0.00
CMA5
2012
2013
2014
2015
2016
2017
2018
2019
Year
Baseline dataset
Corrected dataset

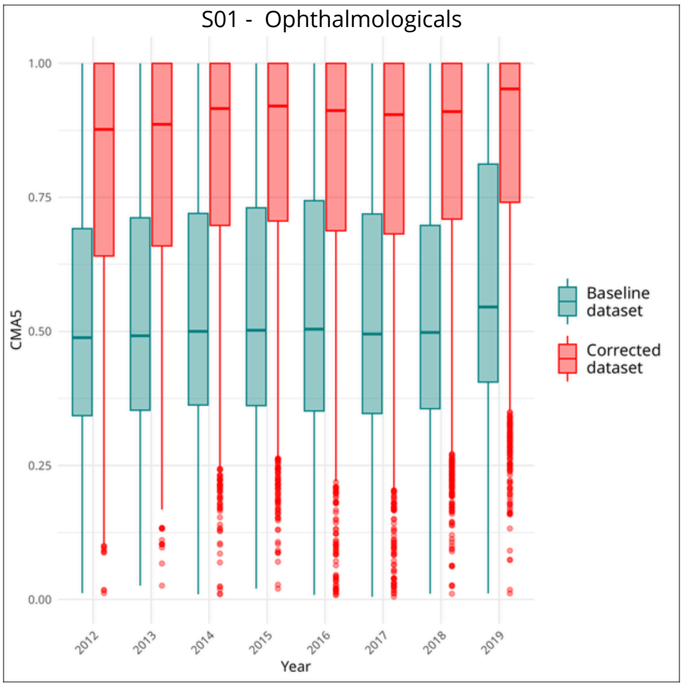
S01 - Ophthalmologicals
1.00
0.75
0.50
0.25
0.00
CMA5
2012
2013
2014
2015
2016
2017
2018
2019
Year
Baseline dataset
Corrected dataset
