# Supplementary material for: A Comprehensive Approach to Days’ Supply Estimation in a Real-World Prescription Database: Algorithm Development and Validation Study
Source: Online J Public Health Inform. 2026 Feb 11;18:e83465. doi: 10.2196/83465 (PMC12936656; doi:10.2196/83465)
Supplement: Multimedia Appendix 2 [file ojphi_v18i1e83465_app2.pdf]

Appendix 2. Prescription distribution by drug groups and days' supply establishing methods, for the full dataset (2012–2019).

DRUG GROUP	DATASET INFORMATION	METHOD TO FINDING DAYS' SUPPLY	PRESCRIPTIONS IN MAIN GROUP
Tablets, capsules, suppositories	Daily dose and days' supply provided	Calculated using given daily dose	398 813 (64.57%)
		Days' supply was used	859 896 (13.92%)
	Only days' supply provided	Days' supply was used	73 814 (11.95%)
	Only daily dose provided	Calculated using given daily dose	2 429 804 (39.34%)
	Daily dose nor days' supply provided	Calculated using imputed daily dose value	2 402 392 (38.90%)
	Days' supply could not be calculated nor imputed		11866 (0.19%)
Semisolid dosage forms	Days' supply provided	Days' supply was used	119 748 (37.55%)
	Days' supply not provided	Imputed as 30 days per package[a]	199 154 (62.45%)
Medicinal nail polish	Days' supply provided	Days' supply was used	3 008 (41.55%)
	Days' supply not provided	Imputed as 210 days or 180 days[a]	4231 (58.45%)
Ear drops	Days' supply provided	Days' supply was used	5 661 (57.11%)
	Days' supply not provided	Imputed as 7 days per prescription[a]	4 252 (42.89%)
Eye drops	Eye drops in single dose containers - daily dose and days' supply provided	Calculated using given daily dose	1008 (0.35%)
		Days' supply was used	637 (0.22%)
	Eye drops in single dose containers - Only days' supply provided	Days' supply was used	46 (0.02%)
	Eye drops in single dose containers - Only daily dose provided	Calculated using given daily dose	5 716 (2.00%)
	Other eye drops - days' supply provided	Days' supply was used	61 649 (21.57%)
	Other eye drops - days' supply not provided	Imputed as 14[a] or 30[b] days per package	216 740 (75.82%)
	Days' supply not calculated nor imputed		43 (0.02%)
Oral drops	Daily dose and days' supply provided	Calculated using given daily dose	33 423 (32.05%)
		Days' supply was used	32 278 (30.95%)
	Only days' supply provided	Days' supply was used	777. (0.75%)
	Only daily dose provided	Calculated using given daily dose	18 662 (17.90%)
	Daily dose nor days' supply provided	Imputed as 30 days per prescription[a]	12 961 (13.20%)

	Days' supply not calculated nor imputed		120 (0.12%)
Inhalation medication	Daily dose and days' supply provided	Calculated using given daily dose	6060 (4.79%)
		Days' supply was used	10 107 (7.98%)
	Only days' supply provided	Days' supply was used	9 778 (7.72%)
	Only daily dose provided	Calculated using given daily dose	72 481 (57.26%)
	Days' supply not provided	Imputed as 60 days per prescription[a]	27 137 (21.44%)
	Days' supply not calculated nor imputed		1 025 (0.75%)
Nasal sprays	Anti-fungal nasal spray - days' supply not provided	Imputed as 7 days per prescription[a]	7 738 (8.72%)
	Daily dose and days' supply provided	Calculated using given daily dose	2 959 (3.33%)
		Days' supply was used	25 517 (28.75%)
	Only days' supply provided	Days' supply was used	23402 (26.37%)
	Only daily dose provided	Calculated using given daily dose	15034 (16.94%)
	Daily dose nor days' supply provided	Imputed as 30 days per prescription[a]	14 094 (15.88%)
Syrups	Antibiotics - days' supply not provided	Imputed as 14 days per prescription[a]	29 130 (34.31%)
	Daily dose and days' supply provided	Calculated using given daily dose	9 023 (10.63%)
		Days' supply was used	996 (1.17%)
	Only days' supply provided	Days' supply was used	2098 (2.47%)
	Only daily dose provided	Calculated using given daily dose	28 228 (33.24%)
	Days' supply not provided	Imputed as 30 days per package[b]	15 436 (18.18%)
Transdermal patch	Hormonal patch - days' supply not provided	Impute as 7 days per patch plus 7 days[a]	13 131 (81.18%)
	Analgesic patch - days' supply not provided	Imputed as 3 days per patch[a]	1 127 (7.01%)
	Days' supply provided	Days' supply was used	599 (5.94%)
	Days' supply not provided	Impute as 7 days per patch[a]	865 (5.38%)
Intravaginal contraceptives	Days' supply provided	Days' supply was used	1 692 (8.00%)
	Days' supply not provided	Imputed as 30 days per item in package[a]	19 453 (92.00%)
Implants and vaccines	Days' supply provided	Days' supply was used	18 940 (45.2%)
	Days' supply not provided	Imputed as 1 day	22 979 (54.8%)
Insulin injections	Days' supply provided	Days' supply was used	2 601 (3.14%)
	Days' supply not provided	Imputed as 60 days per prescription[a]	80 266 (96.86%)
Other	Days' supply provided	Days' supply was used	26 361 (29.70%)
	Days' supply not calculated nor imputed		40 551 (45.69%)
